# Few layer epitaxial germanene: a novel two-dimensional Dirac material

**DOI:** 10.1038/srep20714

**Published:** 2016-02-10

**Authors:** María Eugenia Dávila, Guy Le Lay

**Affiliations:** 1 Instituto de Ciencia de Materiales de Madrid-ICMM-CSIC, C/Sor Juana Inés de la Cruz, 3 Cantoblanco, 28049-Madrid, Spain; 2 Aix Marseille Université, CNRS, PIIM UMR 7345, 13397, Marseille, France

## Abstract

Monolayer germanene, a novel graphene-like germanium allotrope akin to silicene has been recently grown on metallic substrates. Lying directly on the metal surfaces the reconstructed atom-thin sheets are prone to lose the massless Dirac fermion character and unique associated physical properties of free standing germanene. Here, we show that few layer germanene, which we create by dry epitaxy on a gold template, possesses Dirac cones thanks to a reduced interaction. This finding established on synchrotron-radiation-based photoemission, scanning tunneling microscopy imaging and surface electron diffraction places few layer germanene among the rare two-dimensional Dirac materials. Since germanium is currently used in the mainstream Si-based electronics, perspectives of using germanene for scaling down beyond the 5 nm node appear very promising. Other fascinating properties seem at hand, typically the robust quantum spin Hall effect for applications in spintronics and the engineering of Floquet Majorana fermions by light for quantum computing.

After the first compelling evidences of the synthesis in 2012 of two-dimensional (2D) single-layer silicene, graphene’s silicon cousin, on silver (Ag(111)) and zirconium diboride (ZrB_2_(0001)) metallic substrates^1^,^2^,^3^, followed, just three years later, by the fabrication of silicene-based field effect transistors (FETs) operating at room temperature (RT)^4^,^5^, a tremendous attention has been paid to atom-thin group IV elemental honeycomb materials as alternative Dirac systems to graphene. Going down the column, these are respectively, silicene, reviewed recently^6^,^7^, germanene, just synthesized in the last months on gold^8^, aluminum^9^ and platinum^10^ (111) surfaces (although this has been questioned in the latter case because of the likely formation, instead, of a surface alloy^11^), as well as on Ge_2_Pt clusters^12^ and stanene (also sometimes coined tinene), just realized on Bi_2_Te_3_(111) substrates^13^.

Germanene synthesis has been preceded by the realization of germanane^14^, its fully hydrogenated partner (GeH), by wet chemistry starting from solid calcium digermanide (CaGe_2_), the 2D sheets being further stabilized by methyl replacement of the H termination^15^.

Although just in its infancy, germanene has been already reviewed^16^ because of its extremely appealing properties, among which we will stress a few. As silicene, free standing germanene does not exist, but it is predicted to be a buckled honeycomb 2D Dirac material^17^,^18^ with extremely high mobilities of its charge carriers^19^. The spin-orbit coupling along with the ~0.64 Å buckling opens up a ~24 meV band gap at the Dirac points significantly higher than in silicene (1.55 meV); this, together with the non-trivial topological properties, might result in a quantum spin Hall effect detectable at RT^20^. Externally applied electric fields might help tuning the band gap and can generate topological phase transitions and helical zero-energy modes^21^, while proximity with an s-wave superconductor and photo-irradiation might create a photo-induced topological superconductor hosting controllable Majorana fermions^22^. As such, these fascinating properties offer tantalizing prospects for future applications in electronics, spintronics and quantum computing. Typically, since germanium is currently used as a performance booster in thin FET channels, perspectives of using germanene for scaling down beyond the 5 nm node while increasing the speed and lowering the energy consumption of electronic devices appear very promising.

However, germanene must be created artificially, since no parent crystal like graphite for graphene exist in nature. Hence, this new graphene-like germanium allotrope akin to silicene has been just recently grown by dry epitaxy as diverse reconstructed two-dimensional phases on three different single crystal metallic substrates. These atom-thin sheets, which lie directly on the metal surfaces are likely to lose, because of the interfacial coupling, the massless Dirac fermion character and other unique associated physical properties of free standing germanene, as is often considered for monolayer silicene grown on different metals^23^. Here, we prove that this issue can be circumvented, by synthesizing few layer epitaxial germanene and further demonstrating that it possesses Dirac cones because of reduced coupling. Hence, we introduce few layer germanene amongst the limited-number family of existing 2D Dirac materials^18^, aside of its sibling, multilayer silicene^24^,^25^.

In this work, this finding is established on high-resolution synchrotron-radiation-based photoemission (SR-PES) experiments, scanning tunneling microscopy (STM) observations and low energy electron diffraction (LEED).

## Results

### Synthesis and electronic properties of few layer germanene

We grow *in situ* epitaxial few layer germanene under ultra-high vacuum conditions by evaporating germanium at a typical rate of 1 monolayer per 30 minutes onto a clean Au(111) surface at ~200 °C and annealed it for 10 minutes at ~250 °C. The growth is followed by STM imaging at RT, low energy electron diffraction (LEED) and detailed core-level spectroscopy analysis using tunable synchrotron radiation light.

The electronic structure is obtained through high-resolution (k_x_, k_y_) mapping of the valence bands at low temperature (~15 K) at different energy depths below the Fermi level and cross-sectional vertical cuts, which unambiguously evidence the presence of Dirac cones for few layer germanene.

### Growth morphology and epitaxial relationship

**Figure 1:**
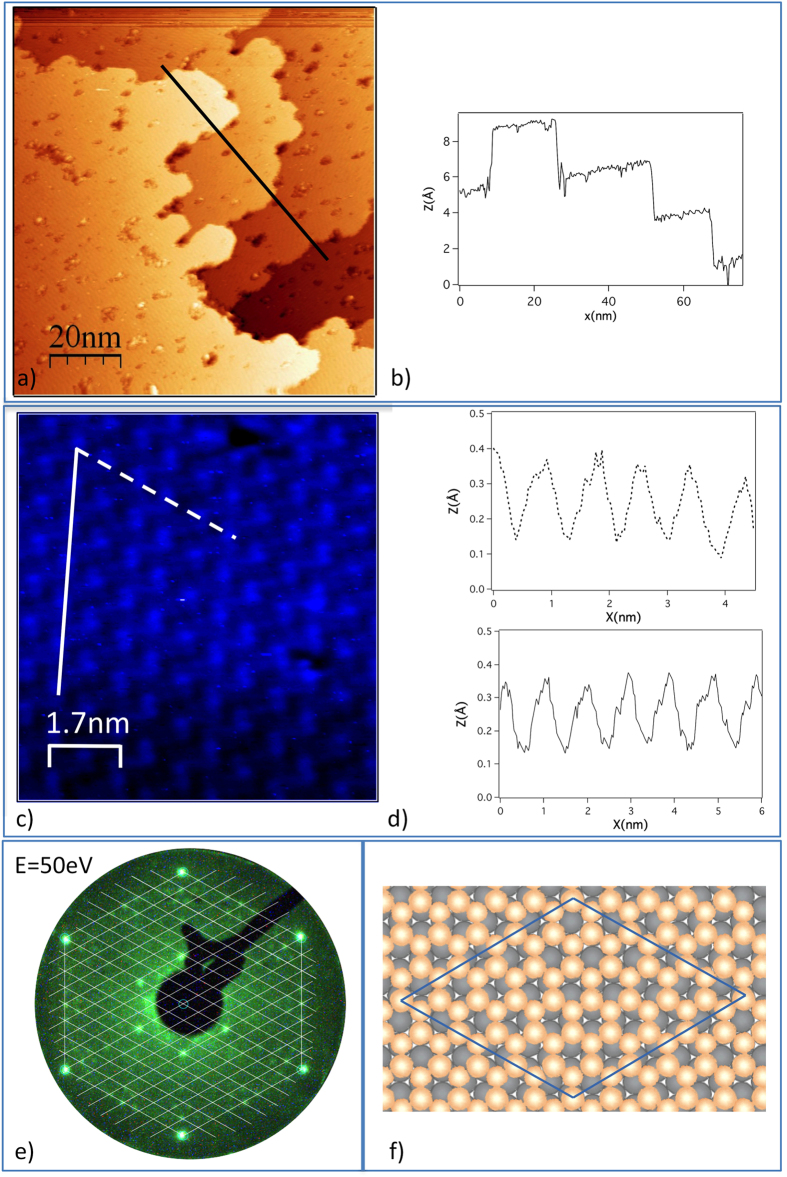
Growth aspects and ball model of few layer germanene. (**a**) Large scale STM image (tunneling current I_t_ = 0.982 nA; sample bias V_s_ = −0.98 V) showing different flat terraces. (**b**) Profile (black line in (**a**)) showing step heights of ~3.2 Å. **(c)** Higher resolution STM topograph (I_t_ = 0.775 nA; V_s_ = −1.1 V) showing large protrusions arranged in a distorted hexagonal arrangement (image as acquired: no drift correction). (**d**) Profiles along the white continuous and dashed lines in (**c**) showing a ~9–10 Å separation between protrusions having heights of just ~0.2 Å. (**e**) Corresponding LEED pattern recorded at 50 eV primary energy (integer order spots are the strong ones at the periphery) superposed with a 8 × 8 grid. (**f**) Top view ball model of epitaxial few monolayer germanene on the Au(111) substrate. For clarity only one germanene layer on top of one Au(111) unreconstructed layer are displayed; the epitaxy is such that a 3√3 × 3√3 reconstructed germanene cell coincides with a 8 × 8 supercell (in blue) of Au(111) with the germanene armchair edges aligned along the dense gold rows.

Past the initial formation of the first 2D monolayer, which presents several co-existing phases^8^, we observe in STM imaging at RT in Marseille, France, large flat terraces extending over several tens on nanometers, separated by steps all about 3.2 Å in height, markedly different from the 2.4 Å height of Au(111) steps, but close to the 3.25 Å step height on Ge(111) surfaces, as shown in Fig. 1 for deposition of about 3 or 4 equivalent monolayers (panels (a) and (b)).

As in all previous studies where silicene forms corrugated reconstructions on metal substrates, but never a primitive 1 × 1 free standing like structure^26^ because of its adaptability linked to the intrinsic buckling, the same being true for monolayer germanene, real atomic resolution could not be achieved. We just observed on those extended terraces large protrusions with ~9–10 Å separations, making it difficult to assign a definite surface cell, but typically just ~0.2 Å in height, as seen in panels (c) and (d). They are seemingly arranged in a distorted hexagonal arrangement since directly acquired STM images are presented without correction for the drift. Indeed, this does not provide any detailed structural information. However, sharp LEED patterns, as displayed in panels (e), with dominating one-eighth order diffraction spots, point essentially to a 8 × 8 supercell with respect to Au(111). Such a large superstructure precludes the formation of a germanium (111) thin film wetted by a gold monolayer, following a surfactant effect^27^, since in such a case just a Ge(111)√3 × √3*R*(30°)-Au reconstruction^28^ (or 4/√3 × 4/√3 with respect to Au(111), as for multilayer silicene on Ag(111)^29^) would be observed.

Inferring, as in all previous situations for silicene and germanene monolayers as well as for silicene multilayers, a hidden underlying honeycomb arrangement, we propose in Fig. 1f a generic top-view ball model where reconstructed few layer germanene sheets in epitaxy would cover the gold surface (for clarity, only one atomic layer of flat germanene on a single unreconstructed Au(111) plane is shown). The epitaxial relation is such that a 3√3 × 3√3 reconstructed germanene cell coincides with a 8 × 8 supercell of Au(111) with the germanene armchair edges aligned along the dense Au rows. The corresponding projected in-plane Ge-Ge interatomic distance would be 2.56 Å, practically identical to that determined for the first layer germanene phase studied by Dávila *et al.*^8^. Here, we recall that for silicene multilayers grown on the archetype 3 × 3 reconstructed first layer silicene phase related to a 4 × 4 Ag(111) supercell, where zig-zag silicene edges coincide with the Ag dense rows, √3 × √3 reconstructed multilayer silicene is formed in a unique orientation^26^,^30^.

For bilayer and multilayer silicene several atomic geometries derived from bilayer and trilayer graphene have been theoretically considered, but an alternate favored arrangement involves so-called dumbbell (DB) units^31^. Although this silicene multilayer DB model still needs to be tested in diffraction experiments, it could be also applicable to few layer germanene^32^. However, we note that the unique in-plane Ge-Ge distance for mono and few layer germanene could possibly point, instead, to a simple A-A stacking as suggested by Acun *et al.*^16^. Indeed, all this indicates the need for further structural studies, which is not surprising since germanene, born just one year ago, is still an infant.

### Refined growth and structural studies through synchrotron radiation shallow core-level spectroscopy

We follow the evolution upon growth at ~200 °C of the Au 4f and Ge 3d spin-orbit splitted shallow core-levels (CLs) of the substrate and the deposit. Their total intensities give a quantitative evaluation of the coverage ratios, while their shape analysis upon thorough program fittings using Doniach-Sunjic line shapes (see the section Methods) provides signatures of the first germanene layer initially formed, and, next, of an interface related component and a developing one beyond one monolayer (ML) coverage.

**Figure 2:**
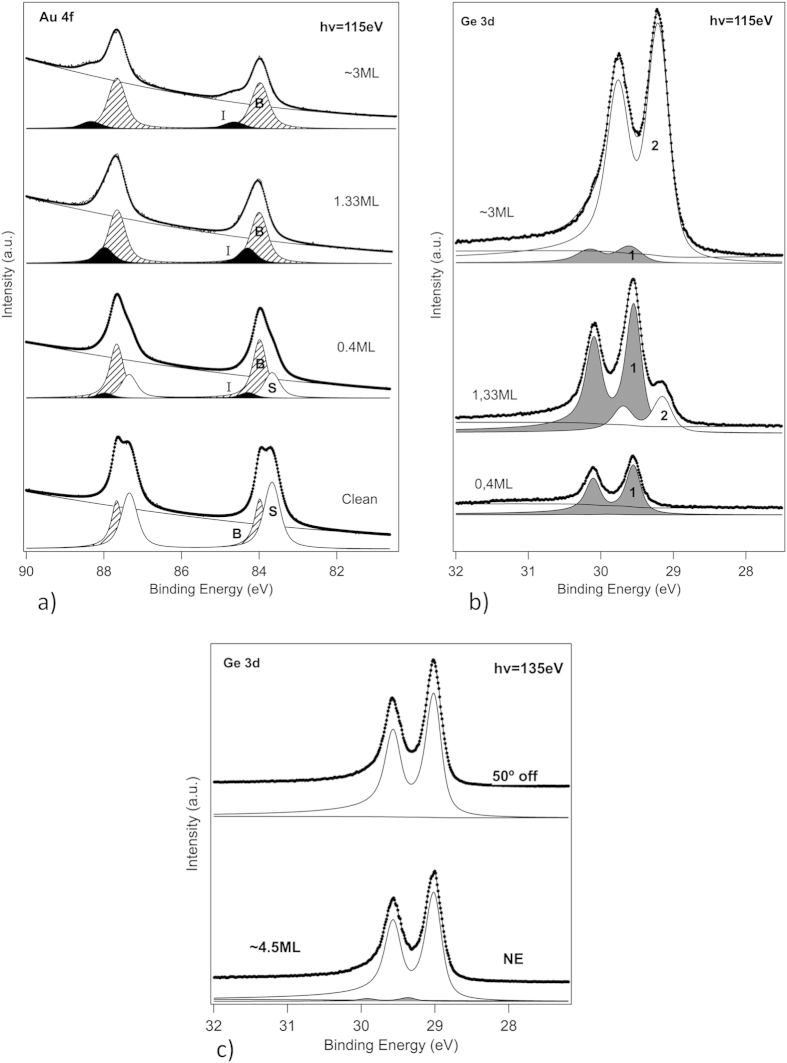
High resolution synchrotron radiation core-level spectroscopy during growth of few layer germanene. (**a**) Normal emission Au 4f shallow core-level (Au 4f_7/2_ and 4f_5/2_ spin-orbit splitted lines) recorded at 115 eV photon energy from the gold substrate at increasing coverages. The spectrum from the initial bare surface displays a strong surface component (S) in addition to the bulk one (B). Upon Ge deposition the (S) component is quenched, while a new component (I) develops at higher binding energy than the bulk one; it is assigned to the interface between the gold substrate and the germanene thin film. (**b**) Same as (**a**) but for the Ge 3d shallow core-level of the growing germanene thin film (Ge 3d_5/2_ and 3d_3/2_ spin-orbit splitted lines). The first germanene layer in contact with the gold substrate gives a high-binding energy component (1), in grey; it is damped beyond the first layer by the development of a second component (2) at lower binding energy. (**c**) Ge 3d core-level from few layer germanene (~4.5 monolayers) at normal and 50° off normal emission recorded at 135 eV photon energy. Comparison of the spectra shows that in very surface sensitive conditions (50° off normal collection angle of the Ge 3d photoelectrons) only one single component is recorded (component (2) in Fig. 2(b)), which further demonstrates that Au atoms do not segregate at the top of the ~4.5 monolayer germanene film.

The evolution from the clean bare Au(111) surface up to ~3 deposited MLs is shown in Fig. 2. The data were acquired at RT at the APE beamline of the Elettra SR facility in Trieste, Italy, at hν = 115 eV photon energy at normal emission (NE). In panel (a) we note the attenuation of the surface component S of the clean Au 4f CL from the initial bare Au(111) surface, totally quenched beyond 1 ML, and the emergence of a new component at higher binding energy (BE ~84.3 eV for the 4f_7/2_ line) than the bulk 4f_7/2_ line B situated at ~84.0 eV. It is related to the top gold layer in contact with the first germanene monolayer^8^. However, it shifts to higher BE, ~84.8 eV, when additional MLs are condensed, while its relative weight is reduced. Clearly the upper gold layer is modified, i.e., a new interface is formed, which has its own signature. This is corroborated in panel (b) where the corresponding Ge 3d CLs at NE (hν = 115 eV) are displayed. A single very narrow Ge 3d component (1) characterizes the first germanene layer^8^ with a ~29.45 eV BE of the 3d_5/2_ line. A somewhat broader, but unique, component (2), shifted by ~0.30 eV to lower BE (29.15 eV), develops from the start of the second layer, while correlatively, the initial component (1) is strongly attenuated. The new developing Ge 3d component is totally different from the line shape components of a clean Ge(111) surface on the one hand, and of the Ge(111)√3 × √3*R*(30°)-Au surface on the other hand^33^. This demonstrates conclusively the formation of a new 2D phase, which we assign to few layer germanene, as will be irrefutably confirmed in the following.

### Electronic structure of few layer germanene: evidence of Dirac cones

For a detailed study of their electronic structure we prepared at the SIS beamline of the Swiss Light Source in Villingen, Switzerland, slightly thicker films (~4.5 MLs). A typical LEED pattern was the one shown in Fig. 1. We first checked the shallow CLs at hν = 135 eV and NE, obtaining similar signatures as those described above, and further collected spectra at 50° away from NE to further increase the surface sensitivity. As shown in Fig. 2(c), the very small initial component seen at NE in the Ge 3d CL spectra taken at RT has totally disappeared at 50° off normal. The reason is that just the film itself is measured because of the reduced probed depth. Clearly, the sharp single component spectra measured in these conditions demonstrate that the film is essentially structurally identical in its “bulk” and at its very surface, which is itself absolutely virgin of any Au atom trace amounts.

### Valence states probed by synchrotron radiation Angle-Resolved Photoemission (ARPES) at ~15 K

**Figure 3:**
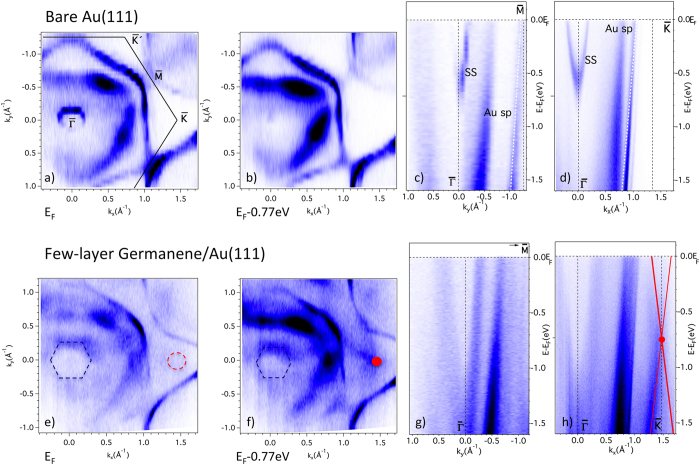
Valence states measured by high-resolution synchrotron radiation ARPES. The top panels (**a–d**) are for the bare Au(111) substrate surface, the bottom ones (**e–h**) for the ~4.5 monolayer germanene thin film; data are collected at 70 eV photon energy. (**a**) Fermi map of the clean Au(111) surface with superposed scheme of the first Brillouin zone with its high symmetry points; the circular cross-section of the Shockley surface state is clearly visible around the zone center 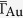. (**b**) Intensity map, i.e., (k_x_, k_y_) horizontal section, at 0.77 eV below the Fermi level; the map being taken below its bottom, the Shockley surface state is no longer recorded. (**c**) Band dispersion E(k_y_) (vertical section) along the 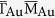 direction; the Shockley surface state SS as well a gold band (Au sp) are indicated. (**d**) Band dispersion E(k_x_) (vertical section) along the 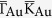 direction; the Shockley surface state SS as well a gold band (Au sp) are indicated. (**e**) Fermi map of the thin germanene film: a new state around the zone center 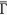 with hexagonal cross-section (dashed blue hexagon superposed) has replaced the initial Shockley surface state of the bare Au(111) substrate surface with circular cross-section in **(a)**; furthermore, a new weak intensity (marked with a red circle) is detected around the 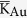 point. (**f**) Intensity map, i.e., (k_x_, k_y_) horizontal section, at 0.77 eV below the Fermi level, as in (**b**): the hexagonal cross-section of the new state is still recorded as in **(e)**, but with a reduced size; the new state (marked with a red circle as in (**e**)) around the 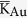 point is now recorded with a strong intensity. **(g)** Band dispersion E(k_y_) (vertical section) along the 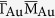 direction as in (**c**); the Shockley surface state SS has disappeared. **(h)** Band dispersion E(k_x_) (vertical section) along the 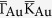 direction as in (**d**); the Shockley surface state SS has disappeared, while new cone like dispersing bands are recorded. Continuous red lines are superposed for clarity and a red dot is placed at the position of the cross-section map in (**f**).

We compared in the same experimental conditions the valence states of the clean, bare Au(111) substrate and those of the ~4.5 ML films, as prepared and described above. To this end we collected full data sets in k space at different photons energies with an angular resolution of 0.5° this correspond to an error of just ±0.05 Å^−1^ on the k_//_ wave vector. We present in Fig. 3 data acquired at hν = 70 eV photon energy. This set covers a large part of the surface Brillouin zone (BZ) and extends over a significant portion of the second BZ. The top panels are for the clean bare Au(111) surface, while the bottom panels are for the ~4.5 ML film.

Panel (a) is the Fermi map, i.e., photoelectron intensity data collected in the (k_x_, k_y_) plane in a narrow interval of ±10 meV around the Fermi energy E_F_; for clarity, the surface BZ is superimposed. Panel (b) is a similar map as in panel (a) but taken at 0.77 eV below E_F_, while panels (c) and (d) show the valence bands dispersions along the high symmetry directions 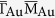 and of the 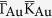 of the surface BZ for the bare Au(111) sample. The sharp Shockley surface state, a paraboloid around the zone center 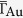 with its bottom at ~0.80 eV below E_F_, characterizes a very clean surface^34^. It is totally quenched, but replaced by a larger cone-like feature with its bottom at ~1.1 eV below E_F_ and a cross-section indicative of hexagonal warping, as for the √3 × √3 silicene phase on Ag(111)^35^, in the corresponding panels (e), (f), (g) and (h) in the presence of the film. One notes in (e) and (f) at the position of the 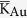 and 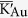 points (void of any filled state for the bare Au(111) surface), new states with a roughly circular cross-section. They are further seen and their dispersion measured along the along 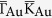 in panel (h). Probably due to matrix elements effects they are hardly detected along the 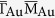 direction in panel (g).

### Experimental evidence of Dirac cones

**Figure 4:**
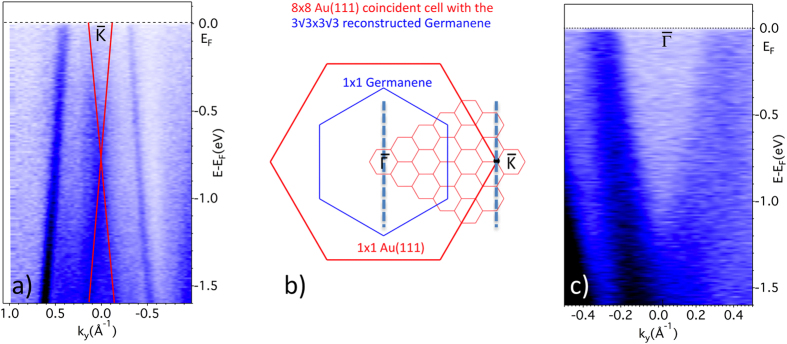
Dirac cones at the 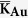 points and at the zone center 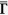. **(a)** Cone-like dispersion, indicated by the red lines, along the top dashed line in panel (**b**) of the new state detected in Fig. 3(h) at the initial position of the 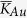 point. (**b**) Scheme of the surface Brillouin zones: the small BZs in pink are common to the 3√3 × 3√3 reconstructed germanene and the 8 × 8 Au(111) coincidence supercell; the 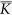 points of these small BZs coincide with the 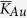 points, while the K and K′ points of the germanene 1 × 1 surface BZ fold at 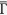 points of the small BZs and, hence, at the main zone center 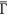. (**c**) Cone-like dispersion, measured along the middle dashed line in panel (**b**) of the new state around the zone center with hexagonal warping, as seen in Fig. 3(e,f); only the upper cone is clearly seen, the bottom one being obscured by strong overlapping Au states.

The new features at the positions of the 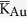 and 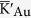 points possess the characteristic signature of a Dirac cone, as displayed in Fig. 4. Panel (a) shows the dispersion around 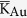 along the direction (parallel to 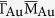) indicated in the BZ scheme of panel (b) where the surface BZ for a 1 × 1 germanene film oriented on a 1 × 1 Au(111) surface according to the real space ball model of Fig. 1f is presented. For the considered 3√3 × 3√3 reconstructed few layer germanene film in coincidence with the 8 × 8 Au(111) supercell the small zones resulting from the reconstruction are also indicated. In (a) two linearly dispersing bands forming a vertical section of Dirac cones cross at ~0.77 eV below E_F_. One notices in (b) that the 1 × 1 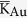 and 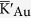 points are actually also 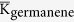 and 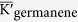 points of the small BZs. The coincidence of these high symmetry points both for the reconstructed BZs of the germanene film and the surface BZ on the gold substrate would make possible the detection of the π and π* germanene states.

The location of the Dirac points at 0.77 eV below E_F_ shows that few layer germanene on Au(111) is n-doped as a result of charge transfer from the gold substrate to the few layer germanene film. A similar situation was already met for multilayer silicene on Ag(111)^24^. The derived Fermi velocity of ~8 × 10^5^ ms^−1^ is remarkably high, surprisingly close to that expected for single layer free standing germanene.

Panel (c) shows the dispersion along the 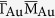 direction of the new, hexagonally warped, π* cone at the zone center 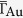. As mentioned above, as a result of charge transfer from the gold substrate, the Dirac point would be at ~1.1 eV below E_F_, but, below, the presence of bands from the gold substrate obscures the detection of the π germanene states. Still, we can derive a Fermi velocity of ~1.1 ×10^6^ ms^−1^ even higher than that measured at the 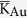 point.

We note that this zone center Dirac cone results only from the few layer germanene film since the two inequivalent 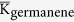 and 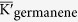 Dirac points in the BZ of the 1 × 1 germanene unit cell are folded at a 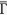 point in the BZ of the reconstructed 3√3 × 3√3 cell. It is the coexistence of these 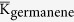 and 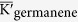 points at such 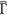 points which results in the hexagonal warping^36^.

## Discussion

### Origin of the Dirac cones

Because of the growth morphology of the grown germanene film (~4.5 MLs) in large, successive, terraces on top of the initial monolayer directly in contact with the Au(111) substrate, some areas, 2, maybe even 3 MLs thick are still not totally decoupled from the gold template. This proximity effect in conjunction with the coincidence of the high symmetry 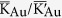 points of the Au(111) surface BZ, void of gold states, with the those of the small germanene BZs, due to the reconstruction, favors the synergetic emergence of Dirac cones at the 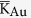 and 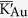 points, with enough intensity so as to be detectable in our ARPES synchrotron radiation measurements.

However, no other Dirac cone is detected at any of the 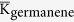 and 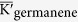 corners of the small BZs, neither, we stress, at the corners of the germanene 1 × 1 BZ itself. Indeed, possibly, their detection could be prevented by matrix element effects and/or the presence of gold bands. However, we believe that a much more fundamental reason explains their absence.

We think that, *per se*, epitaxial 3√3 × 3√3 reconstructed few layer germanene is intrinsically a two-dimensional topological insulator with a single Dirac cone at the zone center. Indeed, free standing single layer germanene itself, like standalone silicene, would be a topological insulator due to the strong spin-orbit interaction and the intrinsic buckled geometric structure^37^,^38^. Even though the exact atomic structure of the 3√3 × 3√3 reconstructed few layer germanene film is not yet known (and not easy to calculate *ab initio* because of the large unit cell), we know that it is perfectly well defined because of the narrow, unique, Ge 3d signature in our high-resolution synchrotron radiation core-level spectroscopy measurements. As we can surely assume that possibly the third, and, for sure, uppers layers in the successive terraces are enough decoupled from the gold substrate to manifest their genuine electronic structure, we consider that the remarkable signature of this intrinsic electronic structure is the warped π* Dirac cone at the zone center 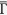. We could measure it in our ARPES experiments because it is partially filled due to of the n-type doping of the film upon charge donation from the gold substrate as demonstrated by the position of the Dirac point at ~1.1 eV below the Fermi level.

These discoveries bring vital perspectives for the possible applications of germanene-based devices. For instance, germanene is found to be a promising material for electronics, spintronics and quantum computing as described bellow.

### Direct applications in electronics

The creation of a two-dimensional Dirac material, a novel germanium allotrope, directly compatible with the existing electronics nanotechnology opens the route to a cornucopia of new potentialities. Typically, as for silicene grown on silver (111) we anticipate transferability of a germanene film grown on a thin epitaxial gold film on mica and encapsulated with a thin Al_2_O_3_ film to, e.g., a silicon wafer with its native oxide^4^, to fabricate germanene based field effect transistors with active channels realized with monolayer, bilayer, and up to few layer germanene thin films. We expect a more facile fabrication of the FETs because germanene will be less reactive than silicene. We further expect initially obtained mobilities superior to the ~100 cm^2^V^−1^.s^−1^ first values achieved with monolayer silicene^4^ in relation with the theoretical higher mobilities calculated for free standing germanene compared to those of standalone silicene^19^. Engineering the band gap of germanene films will not be an issue, as already demonstrated by Goldberger’s group, who has realized hydrogen and methyl terminations by wet chemistry starting from the topochemical deintercalation of the layered van der Waals solid calcium digermanide (CaGe_2_)^14^,^15^.

### Prospects in spintronics and quantum computing

Beyond the practicability of direct integration of germanene in the current electronics industry, one can foresee potential optical applications^39^. The possibility of very high Tc superconductivity should be searched for^40^,^41^,^42^.

Furthermore, the predicted robust 2D topological insulator character, nearly up to room temperature, resulting from the large effective spin–orbit coupling will open the way to the quantum spin Hall effect, the quantum anomalous Hall effect and valley polarized quantum Hall effect in external electric fields^21^,^37^,^43^,^44^,^45^, and, thence, to new fascinating potential applications, especially in spintronics.

Last but not least, proximity with an s-wave superconductor (like lead) will lead to the emergence of Majorana fermions^46^, while photo-irradiation might create a photo-induced topological superconductor hosting controllable Floquet Majorana fermions^22^,^47^, offering tantalizing prospects for quantum computing.

## Methods

### STM observations and LEED patterns

In our work in Marseille, we have studied germanene sheets formed on the Au(111) surface. Clean and well-ordered Au(111) surfaces were prepared by Ar^+^ ion bombardment (1.5 kV, 5 × 10^−5^ mbar) and subsequent annealing of (111)-oriented Au single crystals under ultrahigh vacuum conditions. The *in situ* cleaned Au(111) surface is characterized by its well-known 22 × √3 herringbone structure^48^. We used a germanium evaporator to deposit Ge atoms onto the clean Au(111) surface held at ~200 °C.

Low energy electron diffraction observations and STM measurements in constant-current mode using an Omicron VT-STM with an electrochemically etched tungsten tip were performed at room temperature at different stages of the growth.

### Core-level spectroscopy measurements

We reproduced the sample preparation performed in Marseille at two synchrotron radiation facilities. The reproducibility of the observed LEED patterns at the three different locations with 3 different set-ups served as a unifying reference. The photoemission experiments were first performed on the Surface/Interface Spectroscopy (SIS) X09LA beamline at the Swiss Light Source, Paul Scherrer Institut, Villigen, Switzerland, then repeated (with confirmation) at the APE beamline of the Italian synchrotron radiation facility, Elettra in Trieste. Typically, at the SLS, the beamline was set to linear polarized light. Different photon energies were used; the photo-emitted electrons were collected using a VG‐Scienta R4000 electron analyzer. Here, we present ARPES data recorded at low temperature (about 15 K) with hν = 70 eV and a total energy resolution of 60 meV, as well as shallow Ge 3d core-level spectra acquired at RT at 135 eV photon energy with 80 meV total resolution. The binding energy scale was calibrated with a copper reference sample in direct electrical and thermal contact with the sample. The base pressure of the UHV systems was below 5 × 10^−11^ mbar during the entire measurement and no sign of C and O contaminants was verified by *in situ* X-ray photoelectron spectroscopy (XPS), while no sample and/or data quality degradation was observed. Our results were reproduced on several occasions, using different sample preparations and also in different UHV chambers. As shown in the Supplementary Information different samples grown under similar conditions display the same electronic structure, which confirms the reliability of the experimental data.

### Data analysis

The ARPES data were analyzed using the user friendly software developed by J. Osieki^49^. For the core-level analysis we performed curve-fittings using Doniach-Sunjic lineshapes on integrated backgrounds with typical parameters (Lorentzian and Gaussian Full widths at half maximum, asymmetry parameters) as in ref. 8.

## Additional Information

**How to cite this article**: Dávila, M. E. and Le Lay, G. Few layer epitaxial germanene: a novel two-dimensional Dirac material. *Sci. Rep.* **6**, 20714; doi: 10.1038/srep20714 (2016).

## Supplementary Material

Supplementary Information
