# Supplementary material for: Few layer epitaxial germanene: a novel two-dimensional Dirac material
Source: Sci Rep. 2016 Feb 10;6:20714. doi: 10.1038/srep20714 (PMC4748270; doi:10.1038/srep20714)
Supplement: Supplementary Information [file srep20714-s1.doc]

**Few layer epitaxial germanene: a novel two-dimensional Dirac material**

María Eugenia Dávila^1*^ and Guy Le Lay^2*^

^1^Instituto de Ciencia de Materiales de Madrid-ICMM-CSIC, C/Sor Juana Inés de la Cruz, 3 Cantoblanco, 28049-Madrid, Spain.

^2^Aix Marseille Université, CNRS, PIIM UMR 7345, 13397, Marseille, France.

*Corresponding authors : [mdavila@icmm.csic.es](mailto:mdavila@icmm.csic.es) ; [guy.lelay@univ-amu.fr](mailto:guy.lelay@univ-amu.fr)

**Supplementary information**

**Electronic structure measurements**

Here, we present experimental details during the high-resolution Scanning Tunneling Microscopy and synchrotron radiation core-level spectroscopy studies of Germanene grown *in situ* on Au(111).

Samples were prepared *in situ* in two separate ultrahigh vacuum (UHV) systems, i.e. one with Low Energy Electron Diffraction and STM and one with LEED and Angle Resolved PhotoElectron Spectroscopy (ARPES). STM images were recorded at room temperature using an Omicron variable temperature STM at Aix-Marseille University and the ARPES experiments were first performed on the Surface/Interface Spectroscopy (SIS) X09LA beamline at the Swiss Light Source, Paul Scherrer Institut, Villigen, Switzerland, then repeated (with confirmation) at the APE beamline of the Italian synchrotron radiation facility, Elettra in Trieste. At the SLS (data presented here), to characterize the electronic structure, the beamline was set to linearly polarized light with a photon energy, hν = 70 eV with an energy resolution of 60meV; data were acquired at Low Temperature using a VG-Scienta R4000 electron analyzer using an motorized manipulator where we set a angular step of 0.5°; this correspond to an error of ± 0.05 Å^-1^ on the k// wave vector. The binding-energy scale was calibrated with a copper reference sample in direct electrical and thermal contact with the film. The base pressure of the UHV systems was below 5×10^−11^ mbar during the entire measurement and no sign of sample and/or data quality degradation was observed. Our results were reproduced several times, using different samples grown under the same conditions every 7-8 hours. A single crystal Au(111) substrate was cleaned in vacuum by 1.5-keV Ar ion sputtering for 30 min at 5x10^−5^ mbar. Subsequently, annealing in vacuum at 500° C for 30 min was performed to cure Ar ion sputtering damage and obtain flat and well-ordered surface. The annealing-sputtering cycle was repeated as many times as necessary to obtain a clean surface free of C and O contaminants as verified by *in situ* x-ray photoelectron spectroscopy (XPS). Here, some 4-5 layers of Ge were deposited on the substrate at 200° C by a resistance heated crucible resulting in sharp LEED patterns. A series of experiments was undertaken to determine the rate of germanium evaporation in vacuum as a function of temperature.

Same type of experiments were also performed at the APE beamline of the Italian synchrotron radiation facility, Elettra in Trieste to characterize again the electronic structure. The beamline was set to linear polarized light with a photon energy, hν = 85eV with an energy resolution of 110meV; the data were acquired at Room Temperature using a SCIENTA SES2002 analyzer and we set a manipulator angular step of 0.5°; this correspond to an error of ± 0.04 Å^-1^ on the k// wave vector.

To show the reproducibility of the ARPES data and the evidence of the Dirac cones, we display below the cut at the
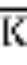
Au point for the preparations recorded at the SIS at the SLS synchrotron at Low Temperature (about 15K) and 70eV and the one at the APE beamline of the ELETTRA synchrotron, recorded at room temperature and 85 eV.


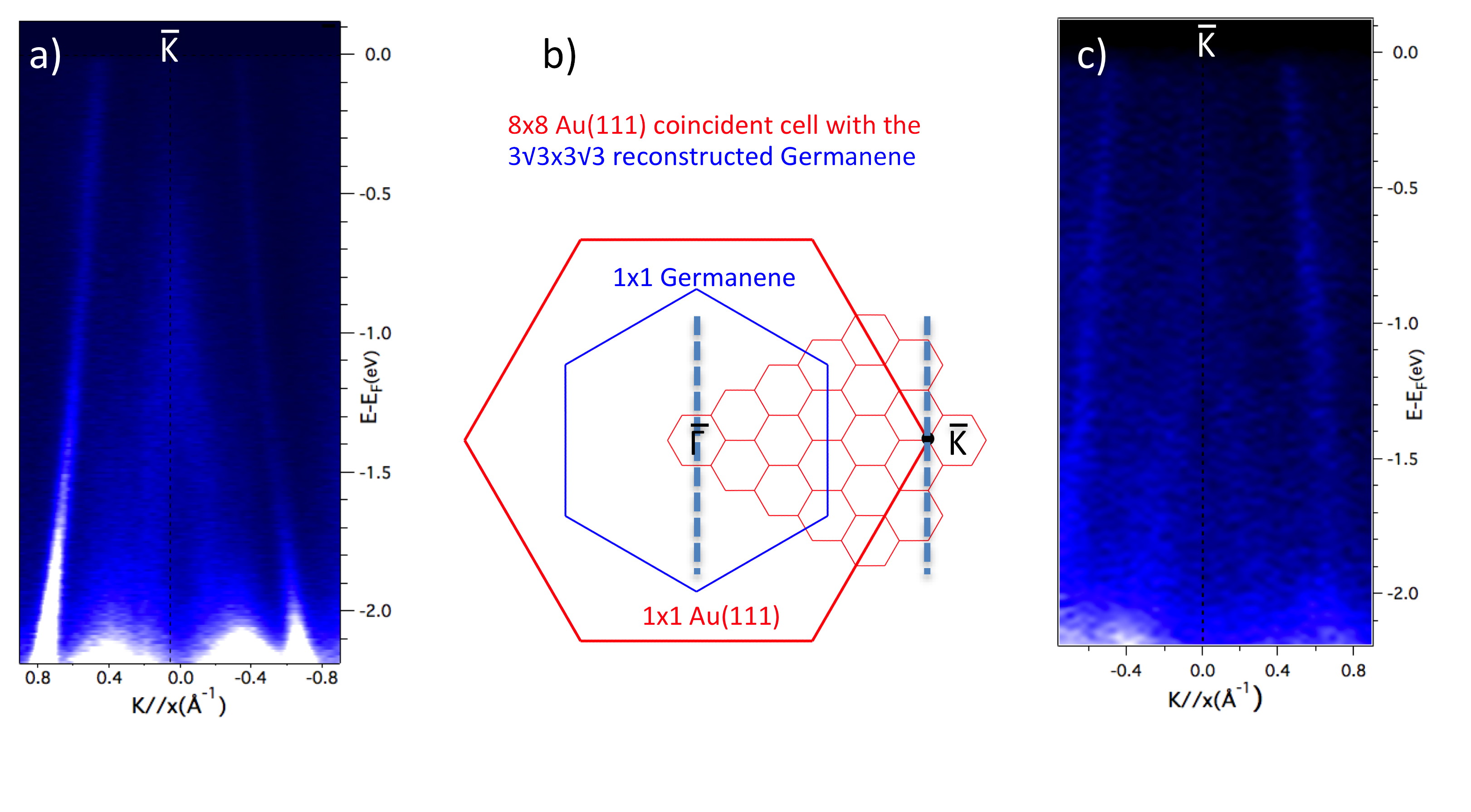


**Dirac cones at the**
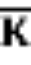
**Au points. (a)** Cone-like dispersion, recorded at SLS synchrotron at LT (15K) and 70eV with a energy resolution of 60meV, along the top dashed line in panel (b) of the new state detected at the initial position of the
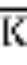
Au point. **(b)** Scheme of the surface Brillouin zones: the small BZs in pink are common to the 3√3 × 3√3 reconstructed germanene and the 8 × 8 Au(111) coincidence supercell ; the
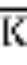
 points of these small BZs coincide with the
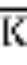
Au points, while the K and K’ points of the germanene 1 × 1 surface BZ fold at
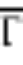
 points of the small BZs and, hence, at the main zone center
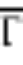
. **(c)** Same cone-like dispersion, recorded at Elettra synchrotron at RT and 85eV with a energy resolution of 110meV, along the top dashed line in panel (b) of the new state detected at the initial position of the
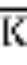
Au point.
